# Low-tidal volume ventilation as compared with conventional tidal volume ventilation in patients of sepsis: a randomized controlled trial

**DOI:** 10.1186/cc11702

**Published:** 2012-11-14

**Authors:** A Waheed, S Bhat, V Nabi, S Gurcu, A Maqbool, S Patigaroo, N Mehfooz

**Affiliations:** 1 Sheri Kashmir Institute of Medical Sciences, Srinagar, India; 2 Government Medical College, Srinagar, India; 3 Government Medical College, Chest Diseases Hospital, Srinagar, India

## Background

In a surgical ICU, low-tidal volume ventilation was compared with conventional tidal volume ventilation. Low-tidal volume ventilation is preferentially used to provide mechanical ventilation in support of patients with acute lung injury, acute respiratory distress syndrome, and inhalation injury. However, few prospective studies have compared low-tidal volume ventilation with normal tidal volume ventilation in sepsis. The purpose of this study was to prospectively compare the two ventilator modalities in an ICU setting in patients of sepsis.

## Methods

A single-center, prospective, randomized controlled trial, comparing conventional tidal volume (12 ml/kg) ventilation with low-tidal volume ventilation (6 ml/kg) in patients with sepsis. In an ICU at a tertiary care teaching hospital, adult patients ≥18 years of age with documented sepsis requiring prolonged (>24 hours) mechanical ventilation were admitted to the ICU. Subjects were randomly assigned to receive mechanical ventilation either by conventional tidal volume ventilation (*n *= 40) or a low-tidal volume ventilation-based strategy (*n *= 40). The first primary outcome was death before a patient was shifted out of the ICU or breathing without assistance. The second primary outcome was the number of days without ventilator use from day 1 to day 28.

## Results

At baseline, both the traditional ventilation group and the low-tidal volume ventilation group had similar demographics to include median age (interquartile range) (35 years (18 to 65) vs. 33 years (18 to 65), *P *= non significant), weight (64 (50 to 82) versus 62 (53 to 70)). The primary outcome was ventilator-free days in the first 28 days after randomization. Our study revealed no significant difference between the low-tidal volume ventilation and the traditional strategy ventilation groups in mean (± SD) ventilator-free days (12 ± 9 vs. 11 ± 8, *P *= non significant) respectively), although the number of days without ventilator use during the first 28 days after randomization was greater in the low-tidal volume group. Mortality was lower in the group treated with lower tidal volumes than in the group treated with traditional tidal volumes (27.0% vs. 30.8%, *P *= non significant); again the difference was not statistically significant.

## Conclusion

Our study revealed that in patients with sepsis, mechanical ventilation with a lower tidal volume may be beneficial in decreasing mortality and increasing the number of ventilator-free days although the difference was found to be statistically insignificant.

